# Trust in Health Information Sources: Survey Analysis of Variation by Sociodemographic and Tobacco Use Status in Oklahoma

**DOI:** 10.2196/publichealth.6260

**Published:** 2018-02-12

**Authors:** Cati G Brown-Johnson, Lindsay M Boeckman, Ashley H White, Andrea D Burbank, Sjonna Paulson, Laura A Beebe

**Affiliations:** ^1^ Evaluation Sciences Unit Division of Primary Care and Population Health Stanford School of Medicine Stanford, CA United States; ^2^ Department of Biostatistics and Epidemiology College of Public Health University of Oklahoma Health Sciences Center Oklahoma City, OK United States; ^3^ Stanford Health for All Alumni Stanford Prevention Research Center Stanford School of Medicine Stanford, CA United States; ^4^ Oklahoma Tobacco Settlement Endowment Trust Oklahoma City, OK United States

**Keywords:** tobacco use cessation, health communication, trust, social media, health care providers, electronic cigarettes, mass media, radio, television, Oklahoma

## Abstract

**Background:**

Modern technology (ie, websites and social media) has significantly changed social mores in health information access and delivery. Although mass media campaigns for health intervention have proven effective and cost-effective in changing health behavior at a population scale, this is best studied in traditional media sources (ie, radio and television). Digital health interventions are options that use short message service/text messaging, social media, and internet technology. Although exposure to these products is becoming ubiquitous, electronic health information is novel, incompletely disseminated, and frequently inaccurate, which decreases public trust. Previous research has shown that audience trust in health care providers significantly moderates health outcomes, demographics significantly influence audience trust in electronic media, and preexisting health behaviors such as smoking status significantly moderate audience receptivity to traditional mass media. Therefore, modern health educators must assess audience trust in all sources, both media (traditional and digital) and interpersonal, to balance pros and cons before structuring multicomponent community health interventions.

**Objective:**

We aimed to explore current trust and moderators of trust in health information sources given recent changes in digital health information access and delivery to inform design of future health interventions in Oklahoma.

**Methods:**

We conducted phone surveys of a cross-sectional sample of 1001 Oklahoma adults (age 18-65 years) in spring 2015 to assess trust in seven media sources: traditional (television and radio), electronic (online and social media), and interpersonal (providers, insurers, and family/friends). We also gathered information on known moderators of trust (sociodemographics and tobacco use status). We modeled log odds of a participant rating a source as “trustworthy” (SAS PROC SURVEYLOGISTIC), with subanalysis for confounders (sociodemographics and tobacco use).

**Results:**

Oklahomans showed the highest trust in interpersonal sources: 81% (808/994) reported providers were trustworthy, 55% (550/999) for friends and family, and 48% (485/998) for health insurers. For media sources, 24% of participants (232/989) rated the internet as trustworthy, followed by 21% of participants for television (225/998), 18% for radio (199/988), and only 11% for social media (110/991). Despite this low self-reported trust in social media, 40% (406/991) of participants reported using social media for tobacco-related health information. Trust in health providers did not vary by subpopulation, but sociodemographic variables (gender, income, and education) and tobacco use status significantly moderated trust in other sources. Women were on the whole more trusting than men, trust in media decreased with income, and trust in friends and family decreased with education.

**Conclusions:**

Health education interventions should incorporate digital media, particularly when targeting low-income populations. Utilizing health care providers in social media settings could leverage high-trust and low-cost features of providers and social media, respectively.

## Introduction

Analysis of US patient health-seeking behavior online in the 2003 Health Information National Trends Survey (HINTS) noted a “tectonic shift in the ways in which patients consume health and medical information” [1]. This shift has significantly changed the landscape for interventions seeking to change health behavior, which can now utilize a growing number of sources to disseminate information, each with their own associated risks and benefits [2]. Digital health interventions (DHIs) are options that use short message service/text messaging, social media, and internet technology, and they have proven effective in mitigating both negative health habits (ie, smoking [3]) and outcomes (ie, cardiovascular disease [4]). In some cases, DHIs can be less expensive to create [5] and DHI programs may leverage patients’ increasing proactivity in obtaining health information online, but may not be trusted or reach all affected demographic groups [6,7]. Conversely, traditional mass media communication sources (eg, television, radio, newspapers, and billboards) are historically high impact, but they can be expensive and do not necessarily target specific populations [8]. Interpersonal communication standbys (eg, health care providers, family and friends, and health insurers) remain consistently ranked as reliable sources for health information, but they have a more limited reach compared to social and mass media, and may be difficult to quantify and standardize [9,10].

Tailoring health communication to an audience is an accepted best practice for interventions. Message source selection is part of this tailoring; considering the source strategy (online, mass media, and/or interpersonal) is complicated by audience receptivity to these sources. Trust is a key element of message receptivity and, in medical settings, trust has been associated with increased health self-efficacy, treatment adherence, and ultimately more positive overall health outcomes [7,11]. However, trust in a specific source may vary based on factors such as prior experience with the source, sociodemographic background, or health behaviors (eg, tobacco use status) [7].

Despite largely ubiquitous access, the trend toward health information-seeking online and in social media is fraught with barriers, misinformation, and mistrust. The internet may exacerbate health disparities: populations with health disparities face barriers to internet access, including disability, lower socioeconomic status, rural locations, and illiteracy [12,13]. Studies done in 2003 and 2012 found that respondents who were female, younger, had higher income, and were better educated were more inclined to seek health information online, leading to a “digital divide” that correlates with preexisting health disparities [13,14].

In addition to access barriers, online health information is of widely varying quality. It is only peer reviewed in certain settings, for instance when health authorities make available clear and well-sourced information [6]. Online health information can be intentionally or unintentionally inaccurate, contain incomplete information, be outdated, originate from lay sources such as chat rooms, or may even encourage pathological behaviors [6]. An example of the emerging issues online health information has created can be summarized in a 2010 case study of randomly selected Twitter status updates about antibiotics [15]. Although only 1.3% of tweets contained information suggesting antibiotic misuse, these few posts reached more than one million followers [15], the equivalent of the population of Oklahoma City, Oklahoma’s largest metropolis.

As data on effective preventive medicine strategies accumulates, public health agencies are devoting increased attention to well-designed, targeted, and longitudinal multicomponent interventions. The Oklahoma Tobacco Settlement Endowment Trust (TSET) was founded in 2000 with a mandate to prevent cancer and cardiovascular disease in Oklahoma. In 2008, TSET partnered with the Oklahoma State Department of Health to create a multiphase communications campaign to raise awareness on the health hazards of tobacco use and secondhand smoke. During campaign evaluations, TSET included questionnaires on trust by source, with the hope that the data would inform future preventive campaigns.

Oklahoma is of particular interest because it may represent a mix of early- and late-adopter mindsets with respect to emerging technologies. These opposite traits could have conflicting impacts on DHI and mHealth trust and acceptance, as indicated in one study showing an association between “personal innovativeness toward mobile services” and mHealth usage [16]. On the late-adoption side, internet penetration in Oklahoma is still the sixth lowest in the United States, at 67.9% [17]. Social media use, measured in Facebook users, is half that (35%) [17]. Oklahoma also remains a state defined by tobacco use, with comparatively high rates of smoking and smokeless tobacco use (21.1% and 6.9%, respectively) [18,19]. However, with respect to the particular technology of electronic cigarettes (“e-cigarettes”) Oklahoma leads in adoption, and has been identified as the only state planning to avoid taxing and sales licensing for these products [20].

Our aim for this study was to continue exploration of these “tectonic shifts” in health information consumption by focusing on trust in a variety of sources in the context of our state of interest (Oklahoma). Furthermore, analysis by tobacco use status and sociodemographic subpopulation would give us insights into positive strategies for targeted DHI and health behavior change messaging, while helping us avoid the potential pitfall of relying on sources that would not be trusted by populations of interest for tobacco use control or other health behaviors.

## Methods

### Sampling Methods

We gathered cross-sectional survey data as part of the evaluation and monitoring of the Tobacco Stops with Me media campaign conducted by the TSET in the spring of 2015 [21]. This cross-sectional survey (N=1001 Oklahomans) took place between May and June 2015 and was a dual-frame sample with both landline telephone and cellular telephone numbers. Eligibility criteria included Oklahoma residency, English speaking, age 18 to 65 years, and verbal consent. Institutional review board approval was obtained from the University of Oklahoma Health Sciences Center.

### Assessing Trust in Health Information by Source

We surveyed trust in seven common sources of health information: television, radio, internet, social media, health care provider, health insurer, and family/friends using this prompt: “Rate how much you trust each of these sources of information.” Trustworthiness for each source was collected on a five-point scale with 1 being “least trustworthy” and 5 being “most trustworthy.”

Initial data analysis revealed that participants tended to rate media sources (television, radio, internet, social media) as less trusted, and rate interpersonal sources (health care provider, health insurer, family/friends) as highly trusted. This skew left us underpowered to compare all seven sources with full scales. Even reduced and dichotomized scale options did not produce meaningful results across all sources. When we re-examined the literature for context, we were reminded of the fundamental differences between, and theoretically independent nature of, mass media and interpersonal sources [9,22]. Thus, for both practical and theoretical reasons, we chose to dichotomize and analyze mass media and interpersonal sources independently. The mass media cluster was dichotomized as trustworthy/neutral (responses 3-5) or not trustworthy (responses 1-2). The interpersonal cluster was dichotomized as trustworthy (responses 4-5) versus not trustworthy/neutral (responses 1-3).

### Assessing Moderators of Trust in Health Information by Source

We assessed tobacco use status using this prompt: “Do you currently smoke cigarettes/use smokeless tobacco/use electronic cigarettes or vapor devices?” Behavior was collected on a three-point scale as “no,” “some days,” and “every day.” For smokers, readiness to change was assessed using the prompt: “What best describes your intentions regarding smoking cigarettes.” Three stages of readiness were collected with a four-point scale; those who selected “never expect to quit” or “may quit in the future, but not in the next 6 months” were categorized as “precontemplation,” those who selected “will quit in the next 6 months” were categorized as “contemplation,” and those who selected “will quit in the next month” were categorized as “preparation.”

We used SAS PROC SURVEYLOGISTIC on both clusters to model the log odds of a participant responding that a source of information was trustworthy or not trustworthy. We addressed potential confounders with multivariate models that controlled for all participant characteristics related to sociodemographics (ie, gender, race/ethnicity, education, income, children in household) and health behavior (ie, smoking status, e-cigarette use status, smokeless tobacco use status). Because the population of Oklahoma is majority white, race/ethnicity was dichotomized into “white” and “other” to reduce degrees of freedom. All reported results are based on weighted data.

Additionally, for context, we assessed how likely respondents were to “look for information on social media about the dangers of secondhand smoke” and to “look for information on social media for free help to quit using tobacco to share with your friends” on four-point Likert scales (ie, “not at all likely,” “not too likely,” “somewhat likely,” and “very likely”).

## Results

Our sample demographics were largely representative of Oklahoma as a whole based on the 2015 US census (Tables 1 and 2) [23]. When the total sample (N=1001) was weighted, 78.55% (725/991) of respondents self-identified as white, 9.48% (97/991) as American Indian, 7.77% (79/991) as African American, 1.66% (65/991) as Hispanic, and 2.54% (25/991) as another race. Half of respondents were female (50.1%, 540/997). Just over half of respondents had at least some college: 29.2% (369/988) had a college degree, 26.5% (312/988) had some college, and 44.3% (307/988) had a high school or equivalent degree or less. Approximately one-fifth of respondents were defined as low income (≤US $30,000/year: 16.9%, 199/891); 31.5% (249/891) were middle income (US $30,000<US $60,000/year), and 51.6% (443/891) were high income (≥US $60,000/year). The sample was relatively evenly distributed between those with children in the household (46.8%, 420/991) and those without children (53.2%, 571/991). In tobacco status, the sample also generally reflected Oklahoma overall: 22.36% (181/1001) were smokers. Smoker readiness to quit skewed toward not being ready (stage of change precontemplation: 62.2%, 96/167; contemplation: 27.8%, 50/167; and preparation: 10.0%, 21/167). Smokeless tobacco and e-cigarette users were slightly overrepresented at 9% each (8.97%, 66/1000 and 8.68%, 79/998, respectively). Close to half of respondents reported use of social media for tobacco-related health information: mean 40.01% (95% CI 36.19-43.99; 406/991) reported being likely to look for information about the dangers of secondhand smoke, and mean 46.13% (95% CI 42.16-50.10; 443/987) reported being likely to look on social media for free help to help friends quit using tobacco.

Trust in sources was split between media and interpersonal sources. For media sources, 24.0% (232/989) of respondents rated the internet as trustworthy, followed by television (20.9%, 225/998), radio (18.2%, 199/988), and social media (11.3%, 110/991) (Table 1). For interpersonal sources, 80.9% (808/994) of respondents rated “health care provider” as trustworthy, followed by friends and family (54.6%, 550/999), and health insurer (48.3%, 485/998) (Table 2).

**Table 1:** Oklahomans’ trust in health information from mass media sources by demographic and tobacco use variables (survey conducted in spring 2015; N=1001).

Demographics		n (weighted %)		Mass media sources, n (weighted %)							
				Trust in social media		Trust in internet		Trust in radio		Trust in television	
Overall		1001 (100.00)		110 (11.3)		232 (24.0)		199 (18.2)		225 (20.9)	
**Gender^a^**											
	Male		457 (49.94)		43 (10.6)		84 (19.1)		84 (15.8)		84 (16.4)
	Female		540 (50.06)		66 (11.9)		146 (28.7)		114 (20.5)		140 (25.3)
**Race/ethnicity**											
	White		725 (78.55)		66 (10.1)		148 (21.7)		134 (16.9)		148 (19.8)
	Native American		97 (9.48)		14 (18.1)		26 (31.5)		26 (25.1)		30 (29.9)
	African American		79 (7.77)		13 (13.5)		30 (37.7)		19 (23.5)		22 (21.4)
	Hispanic		65 (1.66)		11 (12.1)		18 (25.1)		13 (19.9)		18 (26.0)
	Other		25 (2.54)		4 (15.6)		7 (24.3)		5 (18.1)		5 (16.0)
**Education**											
	High school/GED^b^		307 (44.26)		58 (16.3)		80 (26.6)		63 (17.9)		80 (22.1)
	Some college		312 (26.51)		27 (9.2)		74 (26.7)		61 (19.3)		60 (19.5)
	College degree		369 (29.23)		22 (5.7)		73 (17.5)		73 (18.0)		83 (20.5)
**Annual income (US$)^c^**											
	<30,000		199 (16.91)		36 (20.9)		48 (25.9)		42 (23.9)		58 (27.1)
	30,000<60,000		249 (31.52)		30 (14.4)		75 (31.5)		66 (20.6)		61 (24.7)
	≥60,000		443 (51.56)		29 (6.2)		86 (19.8)		71 (15.1)		86 (17.7)
**Children in household**											
	Yes		420 (46.78)		47 (11.1)		105 (25.3)		91 (19.6)		92 (20.1)
	No		571 (53.22)		61 (11.4)		123 (22.8)		106 (17.1)		131 (21.6)
**Smoking status**											
	Smoker		181 (22.36)		26 (16.4)		33 (23.7)		37 (19.9)		42 (23.6)
	Nonsmoker		820 (77.64)		84 (9.8)		199 (24.1)		162 (17.7)		183 (20.1)
**E-cigarette status^d^**											
	E-cigarette user		79 (8.68)		7 (16.1)		11 (27.0)		10 (16.6)		11 (14.9)
	Nonuser		881 (91.32)		102 (10.8)		219 (23.6)		187 (18.2)		214 (21.5)
**Smokeless status^e^**											
	Smokeless user		66 (8.97)		8 (18.0)		13 (27.2)		12 (15.5)		12 (15.8)
	Nonuser		934 (91.03)		102 (10.6)		219 (23.7)		186 (18.4)		213 (21.4)

^a^Multivariable logistic regression showed differences in gender for social media (*P*=.02), internet (*P*<.001), and television (*P*<.001).

^b^GED: General Education Diploma.

^c^Multivariable logistic regression showed differences in annual income for social media (*P*=.04), internet (*P*=.02), and television (*P*=.02).

^d^Multivariable logistic regression showed differences for radio by e-cigarette use status (*P*=.001).

^e^Multivariable logistic regression showed differences for radio by smokeless tobacco use status (*P*=.045).

**Table 2:** Oklahomans’ trust in health information from interpersonal sources by demographic and tobacco use variables (survey conducted in spring 2015; N=1001).

Demographics			n (weighted %)		Interpersonal sources, n (weighted %)				
					Trust in health insurers		Trust in friends & family		Trust in health care provider
Overall			1001 (100.00)		485 (48.3)		550 (54.6)		808 (80.9)
**Gender^a^**									
	Male	457 (49.94)		195 (40.6)		238 (51.0)		350 (77.0)	
	Female	540 (50.06)		288 (56.0)		310 (58.1)		454 (84.6)	
**Race/ethnicity**									
	White	725 (78.55)		347 (48.1)		385 (52.5)		590 (80.9)	
	Native American	97 (9.48)		47 (48.9)		51 (58.5)		76 (81.9)	
	African American	79 (7.77)		45 (51.8)		51 (65.1)		57 (76.2)	
	Hispanic	65 (1.66)		30 (48.6)		38 (59.1)		57 (86.6)	
	Other	25 (2.54)		12 (44.7)		18 (64.1)		23 (93.2)	
**Education^b^**									
	High school/GED^c^	307 (44.26)		141 (45.0)		185 (59.7)		231 (76.7)	
	Some college	312 (26.51)		149 (51.0)		169 (52.4)		250 (82.3)	
	College degree	369 (29.23)		191 (51.3)		189 (48.5)		319 (86.3)	
**Annual income (US$)**									
	<30,000	199 (16.91)		98 (46.5)		126 (64.7)		140 (70.4)	
	30,000<60,000	249 (31.52)		121 (50.2)		138 (54.9)		202 (81.1)	
	>60,000	443 (51.56)		221 (48.2)		225 (51.5)		379 (84.1)	
**Children in household**									
	Yes	420 (46.78)		202 (46.4)		229 (52.6)		350 (80.6)	
	No	571 (53.22)		279 (50.1)		315 (56.2)		452 (81.3)	
**Smoking status**									
	Smoker	181 (22.36)		66 (40.0)		94 (53.6)		126 (70.7)	
	Nonsmoker	820 (77.64)		419 (50.7)		456 (54.8)		682 (83.8)	
**E-cigarette status**									
	E-cigarette user	79 (8.68)		33 (43.3)		38 (53.0)		59 (72.8)	
	Nonuser	881 (91.32)		451 (48.8)		510 (54.7)		747 (81.7)	
**Smokeless status**									
	Smokeless user	66 (8.97)		29 (40.9)		34 (42.9)		50 (78.0)	
	Nonuser	934 (91.03)		456 (49.1)		515 (55.6)		757 (81.1)	

^a^Multivariable logistic regression showed differences in gender for health insurer (*P*=.001).

^b^Multivariable logistic regression showed differences in education for friends and family (*P*=.04).

^c^GED: General Education Diploma.

Demographic and tobacco use status moderators of trust in sources were determined by multivariate logistic regression, and included all participant characteristics (Tables 3 and 4). Within multivariate analyses, trust differences between men and women were significant for television, internet, and social media. Women were up to two times more likely to rate these sources as trustworthy (eg, internet: OR 2.0, 95% CI 1.4-2.9, *P*<.001), and expressed higher levels of trust for all sources. Income also persisted as a factor significantly differentiating trust for social media (*P*=.04), internet (*P*=.02), and television (*P*=.02). As compared to low-income individuals, middle-income individuals were equally likely to trust social media (OR 1.0, 95% CI 0.6-1.8, *P*=.04), but high-income individuals much less so (OR 0.6, 95% CI 0.4-1.1, *P*=.04). As compared to low-income individuals, middle-income individuals were nearly twice as likely to trust internet (OR 2.0, 95% CI 1.2-3.5, *P*=.02), although high-income individuals were slightly less so (OR 1.3, 95% CI 0.8-2.2, *P*=.02). As compared to low-income individuals, middle-income individuals were slightly more likely to trust television (OR 1.2, 95% CI 0.7-2.0, *P*=.17), but high-income individuals were much less so (OR 0.6, 95% CI 0.4-1.1, *P*=.02).

Although tobacco use was not significantly associated with trust in media sources, trust in radio differed for e-cigarette and smokeless users. E-cigarette users were less trusting of radio than nonusers (OR 0.3, 95% CI 0.1-0.6, *P*<.001). Conversely, smokeless users were more trusting of radio than non-smokeless users (OR 2.1, 95% CI 1.0-4.3, *P*=.045). The trustworthiness of providers did not differ by demographic or health indicators. Perceptions of trustworthiness of family and friends varied significantly by education; trust in these close social ties decreased with higher education.

**Table 3:** Summary of multivariable logistic regression analysis for sociodemographic and tobacco use status variables associated with trust in mass media sources (survey conducted in Oklahoma, spring 2015; N=1001).

Variable		Mass media sources									
		Trust in social media			Trust in internet		Trust in radio			Trust in television	
		OR (95% CI)	*P*	OR (95% CI)		*P*	OR (95% CI)	*P*	OR (95% CI)		*P*
**Gender**											
	Male	ref^a^									
	Female	1.5 (1.1-2.2)	.03	2.0 (1.4-2.9)		<.001	1.2 (0.9-1.8)	.25	1.9 (1.3-2.7)		.001
**Race/ethnicity**											
	White	ref									
	Other	1.1 (0.7-1.7)	.58	1.4 (0.9-2.1)		.15	0.9 (0.6-1.3)	.45	1.0 (0.7-1.5)		.94
**Education**											
	High school/GED^b^	ref									
	Some college	1.0 (0.6-1.5)	.86	1.4 (0.9-2.2)		.28	1.1 (0.7-1.6)	.42	1.1 (0.7-1.7)		.36
	College degree	0.9 (0.6-1.4)	.86	1.4 (0.9-2.1)		.28	1.3 (0.8-2.1)	.42	1.4 (0.9-2.2)		.36
**Annual income (US$)**											
	<30,000	ref									
	30,000<60,000	1.0 (0.6-1.8)	.04	2.0 (1.2-3.5)		.02	1.3 (0.7-2.1)	.30	1.2 (0.7-2.0)		.02
	≥60,000	0.6 (0.4-1.1)	.04	1.3 (0.8-2.2)		.02	0.9 (0.5-1.5)	.30	0.6 (0.4-1.1)		.02
**Children in household**											
	No	ref									
	Yes	1.1 (0.7-1.5)	.78	1.4 (1.0-2.0)		.08	1.2 (0.9-1.8)	.24	0.9 (0.6-1.3)		.52
**Smoking status**											
	Nonsmoker	ref									
	Smoker	0.7 (0.4-1.1)	.11	0.9 (0.6-1.5)		.70	1.1 (0.7-1.8)	.75	0.7 (0.4-1.1)		.12
**E-cigarette status**											
	Nonuser	ref									
	E-cigarette user	1.6 (0.8-3.1)	.21	0.8 (0.4-1.6)		.56	0.3 (0.1-0.6)	.001	0.7 (0.3-1.4)		.27
**Smokeless status**											
	Nonuser	ref									
	Smokeless user	1.1 (0.5-2.2)	.86	1.0 (0.5-2.0)		.95	2.1 (1.0-4.3)	.046	1.1 (0.6-2.2)		.76

^a^Ref: reference group.

^b^GED: General Education Diploma.

**Table 4:** Summary of multiple regression analysis for sociodemographic and tobacco use status variables associated with trust in interpersonal sources (survey conducted in Oklahoma, spring 2015; N=1001).

Variable		Interpersonal sources							
		Trust in health insurer		Trust in friends and family			Trust in health care provider		
		OR (95% CI)	*P*		OR (95% CI)	*P*		OR (95% CI)	*P*
**Gender**									
	Male	ref^a^							
	Female	1.8 (1.3-2.5)	.001		1.3 (0.9-1.9)	.11		1.6 (1.0-2.5)	.06
**Race/ethnicity**									
	White	ref							
	Other	1.1 (0.7-1.7)	.60		1.4 (1.0-2.1)	.08		1.1 (0.7-1.9)	.65
**Education**									
	High school/GED^b^	ref							
	Some college	1.3 (0.8-2.0)	.49		0.7 (0.4-1.0)	.04		1.3 (0.8-2.30)	.40
	College degree	1.3 (0.8-1.9)	.49		0.6 (0.4-0.9)	.04		1.5 (0.8-2.8)	.40
**Annual income (US$)**									
	<30,000	ref							
	30,000<60,000	1.2 (0.7-2.0)	.64		0.8 (0.5-1.3)	.51		1.7 (0.9-3.2)	.16
	≥60,000	1.0 (0.6-1.6)	.64		0.8 (0.5-1.3)	.51		1.8 (0.9-3.4)	.16
**Children in household**									
	No	ref							
	Yes	0.8 (0.6-1.1)	.21		0.9 (0.6-1.2)	.39		0.8 (0.5-1.3)	.44
**Smoking status**									
	Nonsmoker	ref							
	Smoker	0.8 (0.5-1.2)	.27		0.8 (0.5-1.2)	.29		0.6 (0.3-1.1)	.12
**E-cigarette status**									
	Nonuser	ref							
	E-cigarette user	1.0 (0.5-1.9)	.98		1.1 (0.6-2.0)	.85		0.8 (0.3-2.0)	.67
**Smokeless status**									
	Nonuser	ref							
	Smokeless user	1.0 (0.5-2.0)	.90		0.7 (0.4-1.3)	.25		1.3 (0.5-3.0)	.61

^a^Ref: reference group.

^b^GED: General Education Diploma.

## Discussion

In a largely representative survey sample of Oklahomans, we found self-reported trust in interpersonal health information sources was higher than in media sources. But this trust was significantly moderated by sociodemographic factors related to gender, income, and education. Women were on the whole more trusting than men, trust in media decreased with income, and trust in friends and family decreased with education. Additionally, and perhaps unexpectedly considering recent documented associations between smoking status and trust in information source [24], we found no association between smoking and trust in any individual source. Alternative tobacco use status, however, was associated with trust in radio: e-cigarette users were less likely to trust radio and smokeless users were more likely to trust radio.

Although less trusted overall, media sources are inexpensive, standardizable, and scalable, so social media may still be effective in targeted DHIs for lower-income populations. A recent systematic review demonstrated the positive impact of mobile phone-based DHIs on cardiovascular disease in general [4], and on smoking specifically [3]. Health information often needs to be tailored to low-income population; the majority of smokers are lower income in Oklahoma [25], a trend repeated in the rest of the United States and globally. Previous studies have posited that low-income communities may be better positioned to receive social media DHIs because individuals may have mobile phone access to social media even if they do not have access to the internet and social media through personal computers [26]. Our study supported these findings. We found that although overall trust in social media was low (11%), individuals in households making less than US $30,000 yearly were significantly more likely than wealthier individuals to trust social media (21% rated social media as trustworthy). Previous reports on the rates of social media utilization in Oklahoma were modest at 36% [27], but rates are likely to increase in tandem with US trends. Our study found that self-reported rates for tobacco-related health information acquisition on social media were high (40%-46%), perhaps because our sample included representative numbers of low-income individuals.

Another argument for the use of social media in DHIs is its potential for social interaction, a desired attribute of successful interventions [28]. Our study found that interpersonal sources were more trusted than media sources, providers were trusted globally, and low-income respondents were more likely to trust friends and family. Social media for health messaging has been identified as a lower-cost communication tool existing in a framework that facilitates community engagement, personal empowerment, and collaboration [29]. An example of this would be TSET’s tobacco prevention Tobacco Stops with Me campaign, where individuals have been invited to share their own stories through social media.

A next step in creating low-cost, high-trust communication could include utilizing health care providers on social media. Previous research has identified the need for tobacco experts to interact in social media to dispel myths about tobacco [22]. This study supports the potential for qualified individuals to make positive impacts in public health by combining high public trust in their opinions and recommendations with easily disseminated and personalized DHI venues. National Health Institutes could further support expert engagement in social media by specifically funding public education through social media as a low-cost way to reach target audiences.

Finally, a word of warning. Our results document that less-educated and lower-income individuals may be more trusting of, and thus more receptive to, health messages from social media and the internet. Although this finding is encouraging for health educators and interventionists, it also puts these health-disparate groups at risk to accept pseudo-health messages from untrustworthy sources. Indeed, there are already indications that at-risk race-ethnicity groups are more trusting of e-cigarette and tobacco companies, that this trust is associated with greater risk of e-cigarette use, and that social media contains tobacco-promotion marketing accessible to youth [24,30,31].

This study has three limitations. Due to the skew of trust results, particularly for social media and providers, we treated mass media (internet, radio, television, and social media) and interpersonal (providers, insurers, and friends and family) sources differently, limiting our ability to compare across groups. Additionally, specific messages, websites, etc, were not tested, so we do not know exactly what participants had in mind when they rated the trustworthiness of each source. Finally, although we speculate about the potential impact on behavior of delivering health information through different sources, this analysis does not offer data to support connections between source trustworthiness and behavior change.

Overall, this study supports the growing body of evidence documenting the potential for DHIs to impact health outcomes, in this case specifically for lower-income and less-educated individuals who may be more receptive and trusting of social media and internet health messages. On a more basic level, in addition to validating previous studies showing the trustworthiness of health care providers regardless of participant smoking status [32], we have extended analysis of trust by smoking status to other sources, and find no significant differences between smokers and nonsmokers. Instead, differences in trust cluster around socioeconomic factors of income, education, and alternative tobacco use (radio), suggesting that successful DHI strategies should be adapted to novel health promotion areas. By contrast, even if content remains consistent, ideal successful programs should be fully reassessed as they are applied to new communities or socioeconomic groups. As DHI programs are reassessed or developed de novo, a primary recommendation based on our findings is to combine ubiquitous high trust in providers with the reach and potential of social media. As attempted in some smoking cessation social media interventions, such as the Tobacco Status Project [33], incorporating expert provider voices into social media interventions may bolster trust and potential efficacy.

## Abbreviations

DHI: digital health intervention
HINTS: Health Information National Trends Survey
GED: General Education Diploma
Ref: reference group
TSET: Oklahoma Tobacco Settlement Endowment Trust

## References

[1] Hesse BW, Nelson DE, Kreps GL, Croyle RT, Arora NK, Rimer BK, Viswanath K (2005). Trust and sources of health information: the impact of the internet and its implications for health care providers: findings from the first Health Information National Trends Survey. Arch Intern Med.

[2] Coulter A, Ellins J (2007). Effectiveness of strategies for informing, educating, and involving patients. BMJ.

[3] Whittaker R, McRobbie H, Bullen C, Rodgers A, Gu Y (2016). Mobile phone-based interventions for smoking cessation. Cochrane Database Syst Rev.

[4] Widmer RJ, Collins NM, Collins CS, West CP, Lerman LO, Lerman A (2015). Digital health interventions for the prevention of cardiovascular disease: a systematic review and meta-analysis. Mayo Clin Proc.

[5] Michie S, Yardley L, West R, Patrick K, Greaves F (2017). Developing and evaluating digital interventions to promote behavior change in health and health care: recommendations resulting from an international workshop. J Med Internet Res.

[6] Cline RJ, Haynes KM (2001). Consumer health information seeking on the Internet: the state of the art. Health Educ Res.

[7] Sbaffi L, Rowley J (2017). Trust and credibility in web-based health information: a review and agenda for future research. J Med Internet Res.

[8] Wakefield MA, Loken B, Hornik RC (2010). Use of mass media campaigns to change health behaviour. Lancet.

[9] Sharma M, Romas J (2016). Theoretical Foundations of Health Education and Health Promotion.

[10] Leisen B, Hyman MR (2001). An improved scale for assessing patients' trust in their physician. Health Mark Q.

[11] Lee Y, Lin JL (2009). The effects of trust in physician on self-efficacy, adherence and diabetes outcomes. Soc Sci Med.

[12] Eng TR, Maxfield A, Patrick K, Deering MJ, Ratzan SC, Gustafson DH (1998). Access to health information and support: a public highway or a private road?. JAMA.

[13] Cotten SR, Gupta SS (2004). Characteristics of online and offline health information seekers and factors that discriminate between them. Soc Sci Med.

[14] Kontos E, Blake KD, Chou WS, Prestin A (2014). Predictors of eHealth usage: insights on the digital divide from the Health Information National Trends Survey 2012. J Med Internet Res.

[15] Scanfeld D, Scanfeld V, Larson EL (2010). Dissemination of health information through social networks: twitter and antibiotics. Am J Infect Control.

[16] Rai A, Chen L, Pye J, Baird A (2013). Understanding determinants of consumer mobile health usage intentions, assimilation, and channel preferences. J Med Internet Res.

[17] Internet World Stats.

[18] (2012). Centers For Disease Control and Prevention.

[19] Oklahoma State Department of Health.

[20] Alvarado M, King S, Kumar A, Osborne CP, Solomon PJ (2014). Electronic Cigarettes: Secondary Research Overview.

[21] White AH, Brown-Johnson GG, Martinez SA, Paulson S, Beebe LA (2015). Oklahoma “Tobacco Stops with Me” media campaign effects on attitudes toward secondhand smoke. J Okla State Med Assoc.

[22] Brown-Johnson CG, Sanders-Jackson A, Prochaska JJ (2014). Online comments on smoking bans in psychiatric hospitals units. J Dual Diagn.

[23] (2015). United States Census Bureau.

[24] Case KR, Lazard AJ, Mackert MS, Perry CL (2017). Source credibility and e-cigarette attitudes: implications for tobacco communication. Health Commun.

[25] America's Health Rankings.

[26] Anguiano B, Brown-Johnson C, Rosas LG, Pechmann C, Prochaska JJ (2017). Latino adults' perspectives on treating tobacco use via social media. JMIR Mhealth Uhealth.

[27] Chou WS, Hunt YM, Beckjord EB, Moser RP, Hesse BW (2009). Social media use in the United States: implications for health communication. J Med Internet Res.

[28] Chou WS, Prestin A, Lyons C, Wen K (2013). Web 2.0 for health promotion: reviewing the current evidence. Am J Public Health.

[29] Lister C, Royne M, Payne HE, Cannon B, Hanson C, Barnes M (2015). The Laugh Model: reframing and rebranding public health through social media. Am J Public Health.

[30] Alcalá HE, Sharif MZ, Morey BN (2017). Misplaced trust: racial differences in use of tobacco products and trust in sources of tobacco health information. Nicotine Tob Res.

[31] Lau AY, Gabarron E, Fernandez-Luque L, Armayones M (2012). Social media in health--what are the safety concerns for health consumers?. HIM J.

[32] Nelms E, Wang L, Pennell M, Wewers ME, Seiber E, Adolph MD, Paskett ED, Ferketich AK (2014). Trust in physicians among rural Medicaid-enrolled smokers. J Rural Health.

[33] Ramo DE, Thrul J, Chavez K, Delucchi KL, Prochaska JJ (2015). Feasibility and quit rates of the tobacco status project: a Facebook smoking cessation intervention for young adults. J Med Internet Res.

